# Efficiency of tetrofosmin versus sestamibi achieved through shorter injection-to-imaging times: A systematic review of the literature

**DOI:** 10.1007/s12350-020-02093-5

**Published:** 2020-03-31

**Authors:** W. Lane Duvall, James Case, Justin Lundbye, Manuel Cerqueira

**Affiliations:** 1 Hartford Hospital, Heart and Vascular Institute, 80 Seymour Street, Hartford, CT 06102 USA; 2 Cardiovascular Imaging Technologies, Kansas City, MO USA; 3 The Greater Waterbury Health Network, Waterbury, CT USA; 4 Department of Nuclear Medicine, Department of Cardiovascular Imaging, Heart and Vascular Institute, Cleveland Clinic, Cleveland, OH USA

**Keywords:** Tetrofosmin, Sestamibi, SPECT MPI, Efficiency

## Abstract

Based on superior image quality, more accurate gated images, and lower radiation exposure to patients, Technetium-99m (Tc-99m) based tracers are preferred over Thallium-201 for SPECT myocardial perfusion imaging. The two Tc-99m tracers, sestamibi and tetrofosmin, have many similar characteristics but there are differences in blood and liver clearance rates, as well as the recommended time after injection for imaging to achieve optimal image quality. Because published peer-reviewed studies examining optimal times between injection and imaging are limited, it can be difficult to identify evidence-based opportunities to optimize imaging protocols. Using systematic literature review methods, this study was designed to identify and consolidate the available evidence on the use of sestamibi compared to tetrofosmin for variable injection to imaging times in regard to test efficiency, including test length and re-scan rates, and image quality, including overall quality and cardiac to extra-cardiac ratios. The composite of this data shows that earlier imaging with tetrofosmin is equivalent to later imaging with sestamibi when assessing subjective image quality or when quantifying heart-to-extra-cardiac ratios. Image quality and heart-to-extra-cardiac ratios comparing early versus later imaging with tetrofosmin were comparable if not equivalent to each other. The equivalency of the imaging quality occurs with 15 minutes (on average) earlier imaging compared to sestamibi and 30 minutes compared to standard time tetrofosmin. The subjective findings of equivalent image quality are also shown with objective measurements of heart-to-extra-cardiac ratios. In this review, the significantly shorter injection-to-acquisition times with tetrofosmin compared to sestamibi resulted in better efficiency and less waiting times for patients; in addition, significantly higher re-scan rates with sestamibi compared to tetrofosmin due to hepatic activity contributed to better throughput with tetrofosmin.

**Electronic supplementary material:**

The online version of this article (10.1007/s12350-020-02093-5) contains supplementary material, which is available to authorized users.

## Introduction

Based on superior image quality, more accurate gated images, and lower radiation exposure to patients, Technetium-99m (Tc-99m) based tracers are preferred over Thallium-201 for SPECT myocardial perfusion imaging (MPI). Statistics from an International Atomic Energy Agency (IAEA) cross-sectional study demonstrate the vast majority of MPI studies in patients under 70 years of age in the United States (approximately 96%) and in Europe (approximately 95%) are performed with Tc-99m sestamibi, approved by the Food and Drug Administration in 1990, or Tc-99m tetrofosmin, approved in 1996.^1^ Although the two tracers have many similar characteristics, there are differences in blood and liver clearance rates as well as the recommended time after injection for imaging to achieve optimal image quality. The selection of a specific technetium-based agent for SPECT MPI is likely to be based on physician personal preference, knowledge of differing kinetics and properties of the two agents, radiotracer cost, and local availability.

The package labeling for these agents and American Society of Nuclear Cardiology (ASNC) guidelines suggest shorter injection to imaging times for tetrofosmin than sestamibi. The US package labeling of Myoview™ (tetrofosmin, GE Healthcare) states that “imaging may begin 15 minutes after administration of the agent,” while the package labeling of Cardiolite® (sestamibi, Lantheus) does not specify an imaging delay time.^2^,^3^ The ASNC Imaging Guidelines for SPECT Nuclear Cardiology Procedures: Stress, Protocols, and Tracers suggest that “optimal validation of imaging times has not been extensively studied, and factors such as camera availability and the presence of liver and gastrointestinal activity influence the optimal imaging times,” and that “a range of imaging times is suggested.”^4^ For Tc-99m sestamibi, minimum delays of 15 to 20 minutes for exercise, 45 to 60 minutes for rest, and 60 minutes for pharmacologic stress are recommended.^5^ For Tc-99m tetrofosmin, minimum delays of 10 to 15 minutes for exercise, 30 to 45 minutes for rest, and 45 minutes for pharmacologic stress are optimal.^5^

Published peer-reviewed studies examining optimal times between injection and imaging generally focus on a single property of the tracers. Because of this, it can be difficult to identify evidence-based opportunities to optimize imaging protocols. Systematic literature reviews have been used in medical research to investigate relevant questions that may be difficult to examine in a single study. These reviews differ from a conventional literature review in that an unbiased systematic survey is conducted of the relevant literature, thus avoiding author and regional biases in selecting papers to be reviewed.^6^ It also allows for the discovery of peer-reviewed articles that may not be well known to the clinical community.

Using systematic literature review methods, this study was designed to identify and consolidate the available evidence on the use of sestamibi compared to tetrofosmin for variable injection to imaging times in regard to test efficiency, including test length and re-scan rates, and image quality, including overall quality and cardiac to extra-cardiac ratios.

## Methods

A review of the medical literature was conducted with the application of a standard systematic literature review methodology as published by the Cochrane Collaboration,^7^ and in line with the PRISMA guidelines.^8^ Searches were conducted in the Embase® database on 12 October 2018, to include studies on the basis of pre-defined eligibility criteria. All the records retrieved from the literature search (using multi-string search strategy) were screened based on the title and abstract supplied with each citation (Table 1). The keywords used were chosen to evaluate the outcome inclusion criteria listed in Table 1. Diagnostic accuracy between sestamibi and tetrofosmin was not investigated due to small sample size, incomplete data and referral bias on patients with coronary angiography thus preventing a valid comparison.

**Table 1:** Study inclusion and exclusion criteria

	Inclusion criteria	Exclusion criteria
Study population	Adults (≥18 years old) with known or suspected ischemic heart disease (IHD) or coronary artery disease (CAD)	Pediatric population Animal/in-vitro studies
Modality	SPECT MPI	Modality other than reported
Interventions	Tetrofosmin (Myoview^TM^)	Intervention other than reported
Comparators	Sestamibi (Cardiolite®)	No exclusion on comparator
Outcomes	Efficiency/ productivity (performance, yield, output, work rate) Throughput, workflow Image quality (diagnostic accuracy, imaging artifacts, intra-observer agreement, repeated scans/re-imaging) Protocol, acquisition time Dosimetry/ effective radiation dose Liver clearance, gastrointestinal activity, gastrointestinal tracer activity and subdiaphragmatic activity	Outcomes other than reported
Study design	Clinical trials and observational studies (Including both comparative and single arm studies)	Reviews/editorials/letters/comments Case study/case series/case report
Language	English	Studies published in language other than English
Publication date	1996 to present	Studies published before 1996
Country	No limits	

Each citation was screened by two independent reviewers and any discrepancies between reviewers were reconciled by a third independent reviewer. Citations that did not match the eligibility criteria in Table 1 were excluded at this abstract screening stage; unclear citations were included. Duplicates of citations (due to overlap in the coverage of databases) were also excluded at the abstract screening stage. The eligibility criteria were then applied to the full-text citations. Each full-text publication was screened by two independent reviewers and any discrepancies between reviewers were reconciled by a third independent reviewer.

Study quality and applicability were assessed by a modified checklist based on the Quality Assessment Tool for Diagnostic Accuracy guidelines (QUADAS-2).^9^ QUADAS-2 is structured so that four key domains are each rated in terms of the risk of bias and the concern regarding applicability to the research question. Each key domain has a set of signaling questions (yes/no/unclear) to help reach the judgments regarding bias and applicability (low/high/unclear).

## Results

**Figure 1:**
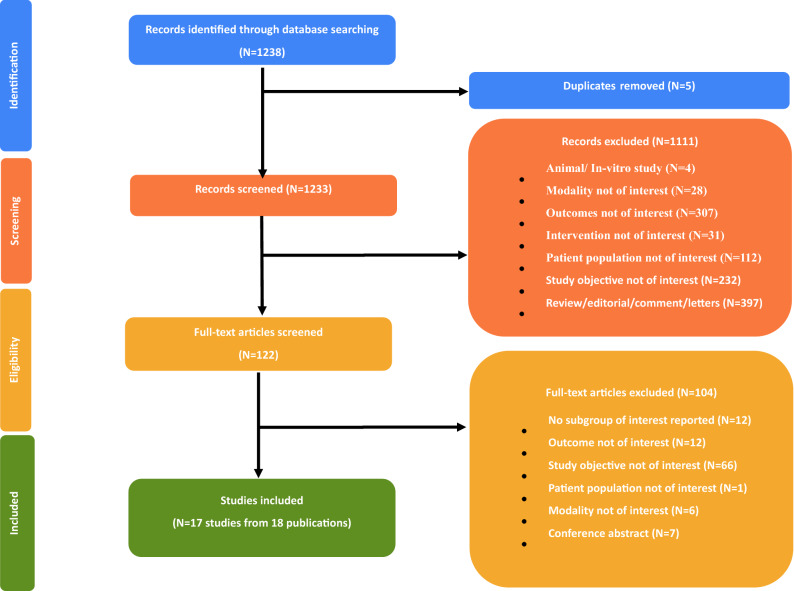
Methodology of the systematic review

A total of 17 studies from 18 publications were included after screening the studies on the basis of pre-defined eligibility criteria (Fig. 1). Of the 17 included studies, the majority (ten) compared early imaging to standard image acquisition time, i.e., injection to imaging time. Nine studies directly compared tetrofosmin and sestamibi, while the remaining eight provided data exclusively for tetrofosmin. A total of 4,835 patients were assessed in the 17 studies identified. The mean age of the patients ranged between 51 and 69 years and it was comparable among the included studies; however, different male/female ratios were seen, with a male predominance (50.5% to 90%) in the majority of studies.

Low risk of bias and lower concerns regarding applicability were observed in the QUADAS-2 tool. This tool uses 4 domains for assessing the risk of bias in a clinical study: patient selection, index test, reference standard and flow and timing.^9^ For example, patient selection for a clinical study should include consecutive or a random sample of eligible patients. However, when arbitrary exclusions are introduced, such as “difficult to diagnose” or other exclusions that would overestimate a particular endpoint, biases are introduced and the value of the publication is diminished. Similar approaches are used for the other three metrics. Across the included studies, most of the studies had low risk of bias in the patient selection domain of the QUADAS-2 tool. Approximately 94%, 65%, 47%, and 65% of studies had a low risk of bias in the patient selection, index test, reference standard, and flow and timing, respectively. The majority of the included studies had low applicability concern of the QUADAS-2 tool. Approximately 88%, 65%, and 47% of studies had a low concern regarding applicability in the patient selection, index test, and reference standard domains of the QUADAS-2 tool, respectively.

### Comparisons of Subjective Image Quality

Subjective semi-quantitative grading of image quality was the most commonly described endpoint in these studies. Image quality is directly related to ease and accuracy of image interpretation. Studies typically employed a 3- or 4-point scale quantifying image quality as assessed by expert reader(s).

#### Comparison of tetrofosmin and sestamibi start times differences: Subjective image quality

One study involving 32 patients who underwent an exercise-rest one-day SPECT MPI study with both tetrofosmin and sestamibi within a week examined image quality between the two tracers.^10^ This was one of the few studies to image with tetrofosmin and sestamibi in the same patient. Imaging with sestamibi was performed 60 minutes after tracer injection while there was only a 30 minute delay with tetrofosmin. Image quality was judged by two independent, blinded readers and graded as high, good, and poor quality. All SPECT images were considered suitable for interpretation without overlapping extra-cardiac activity. There was no significant difference between sestamibi and tetrofosmin in the proportion of high, good, and poor image quality despite the 30 minute shorter imaging time delay with tetrofosmin.

Hurwitz et al examined a small number of tetrofosmin (53) and sestamibi (54) patients over a total of four weeks by alternating the tracer used.^11^ The first two weeks of comparison were performed with “usual” image timing 30 to 60 minutes after stress and the second two weeks with “early” imaging 15 to 30 after stress. Image quality was reviewed on a 1 to 10 scale in a blinded manner. Eighty-one percent of patients underwent dipyridamole stress and only 33% had a one-day study. Overall, sestamibi was rated higher than tetrofosmin in image quality (*P* = 0.04 but values not provided), and the longer imaging delay time was rated higher than the shorter delay for both isotopes combined (*P* = 0.008).

A large, multi-center, randomized study by Kapur et al that included 1,620 patients who received sestamibi or tetrofosmin assessed image quality, attenuation artifact, and low-count rates using a 4-point scale.^12^ For technetium-based tracers a one-day stress-rest protocol was used with stress imaging occurring 30 to 60 minutes after injections and rest images performed 30 to 60 minutes after the rest injection. Upon completion of the study, tetrofosmin imaging was performed on average 10 minutes earlier than sestamibi (40 minutes vs 49 to 50 minutes). Image quality (stress/rest) was not statistically significantly different between sestamibi (2.18/2.39) and tetrofosmin (2.18/2.42). Similarly, there was no significant difference in the number of attenuation artifacts and low-count assessments between sestamibi and tetrofosmin, despite a slightly shorter imaging delay with tetrofosmin.

**Figure 2:**
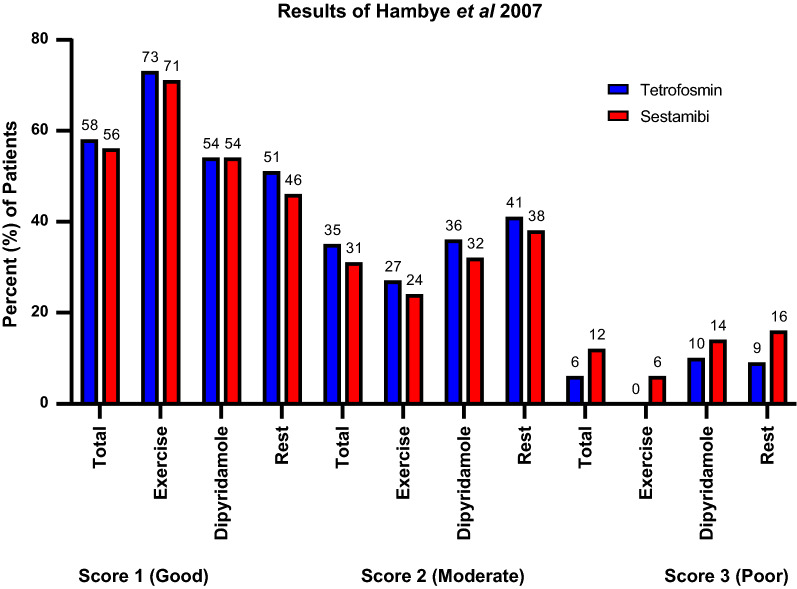
Image quality distribution comparing sestamibi and tetrofosmin based on stressor and rest in Hambye et al evaluating 425 patients.^13^ The injection to imaging times were similar for rest (64 min sestamibi vs 60 min tetrofosmin) and exercise stress (80 min sestamibi vs 86 min tetrofosmin) with imaging after pharmacologic stress occurring 27 min later (at 112 min) for sestamibi compared to tetrofosmin (85 min)

A study by Hambye et al enrolled 425 consecutive patients over four months of weekly alternations between sestamibi and tetrofosmin.^13^ Stressors included exercise or dipyridamole which were similarly proportioned in both groups. There were no planned differences in the injection to imaging times, which was found to be true for rest (64 minutes sestamibi vs 60 minutes tetrofosmin, *P* = 0.48) and exercise stress (80 minutes sestamibi vs 86 minutes tetrofosmin, *P* = 0.42). However, with pharmacologic stress sestamibi imaging occurred 27 minutes later (112 minutes) compared to tetrofosmin (85 minutes; *P* = 0.02). Image quality was judged on a 3-point scale. More tetrofosmin images were found to be of good quality (scores 1 and 2) than sestamibi (93.7% vs. 87.8%) and consequently a higher number of sestamibi images to be of poor quality (score 3) compared to tetrofosmin (12.2% vs. 6.3%) (Figure 2). The authors did report a statistical analysis of this data but did state that they found no significant relationship between the mean time to acquisition and image quality.

Of the four studies examined, 2 of the studies were unable to detect a difference in image quality between the sestamibi based protocols and the tetrofosmin based protocols. Also, in one study tetrofosmin was rated as superior, while in one other study, sestamibi was rated superior (Table 2).

**Table 2:** Studies identified with a total of 3,124 patients assessing image quality comparing sestamibi to tetrofosmin image acquisition, often with the tetrofosmin acquisition occurring early

Author	# of patients	Imaging delay (min)		Sestamibi higher image quality	Equivalent image quality	Tetrofosmin higher image quality
		Sestamibi	Tetrofosmin			
Acampa et al.^10^	32	60	30			
Hurwitz et al.^11^	107	30–60	15–30			
Kapur et al.^12^	2,560	50	40			
Hambye et al.^13^	425	No planned differences				

The size of the diamonds is proportional to the number of patients analyzed in each study

#### Comparison of tetrofosmin start times differences: Subjective image quality

**Figure 3:**
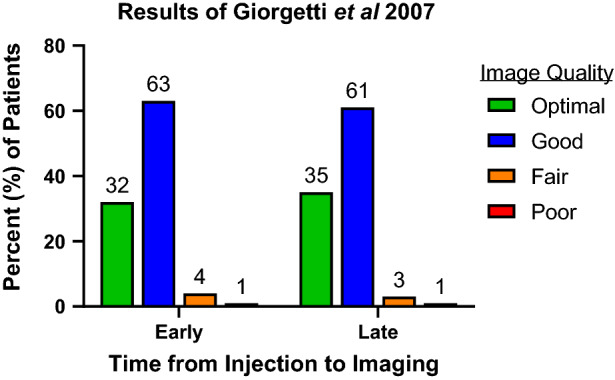
Image quality comparing early versus late imaging of tetrofosmin in 120 patients.^14^*P* value was not statistically significant for all comparisons

In 2007 a study of 120 patients investigated differences in injection to imaging delays with tetrofosmin by acquiring stress and rest images after a short (within 15 minutes) imaging delay followed by images at a traditional (45 to 60 minutes) imaging delay.^14^ Patients underwent either a one-day stress-rest protocol or a two-day protocol with 67% having exercise stress and 33% having dipyridamole stress. Study quality was graded on a four-point scale by two observers and the authors found no statistical difference between acquisition times. There were 114 optimal or good studies at the shorter time and 115 at the longer time and only 1 poor study in each group which was not statistically significant (Figure 3).

A small study enrolled 49 consecutive patients undergoing a two-day exercise or adenosine SPECT MPI study with tetrofosmin where stress and rest images were acquired at 15 minutes and 45 minutes post tracer injection.^15^ Two blinded observers analyzed the studies for liver, intestine and subdiaphragmatic tracer uptake on a four-point scale. Late imaging had better image quality based on lower mean subdiaphragmatic uptake compared to early imaging (At rest: 1.94 vs. 0.56; at stress: 1.04 vs. 0.4). The majority (87.8%) of patients had better quality images at late imaging. The authors also reported differences in the calculated LVEF between early and late imaging due to lack of adequate endocardial border estimation.

In a multi-center registry, Philippe et al imaged 194 patients with tetrofosmin early post-stress and at 30 minutes post-stress, as well as 30 minutes following rest injection.^16^ The exact time of the early post-stress imaging was not defined. A four-point scale of image quality was used as well as a yes or no assessment of subdiaphragmatic activity and the reliability of endocardial edge detection. Similar image quality was seen at early post-stress SPECT (excellent/good in 93.9%) and 30 minute post-stress SPECT (excellent/good in 96.6%) with tetrofosmin. Adjacent subdiaphragmatic activity was seen in 24% of the early acquisitions, 22% of the 30 minute acquisitions, and 31% of rest images which resulted in suitable endocardial border detection in 92% of the early images, 93.7% of the 30 minute images, and 89.5% of the rest images.

A study of 97 patients compared tetrofosmin imaging at two different injection to imaging times of 15 and 45 minutes following adenosine stress using a two-day protocol with a 30 minute delay for rest imaging.^17^ Image quality was again evaluated using a four-point scale as well as extra-cardiac activity. Improved image quality with tetrofosmin was seen in 24% of patients from early to standard imaging, with image quality worsening in 8% patients (*P* = 0.005). There was no statistically significant difference in the proportion of patients who had no subdiaphragmatic activity, but more patients in the early group (18%) had a moderate level of subdiaphragmatic activity compared to the later imaging group (5%; *P* = 0.05).

A smaller but similar study by Dizdarevic et al. enrolled 50 patients undergoing a two-day adenosine stress tetrofosmin MPI study; it also imaged with 15- and 45-minute delays for both stress and rest images.^18^ An almost equal number of early and late images were of optimal quality in this study (92% vs. 96%; *P* = 0.40). No statistically significant differences in LVEF were seen between groups.

Finally, Katsikis et al performed essentially the same study using a one-day stress-rest protocol with exercise or adenosine stress with 78 patients who were imaged with tetrofosmin early and late (15 and 45 minutes) following stress and rest tracer injection.^19^ Image quality was assessed using a four-point scale, and 93% of the early imaging group had optimal or good image quality compared to 98% of the late imaging group. There was no significant difference in the calculated LVEF between groups.

**Figure 4:**
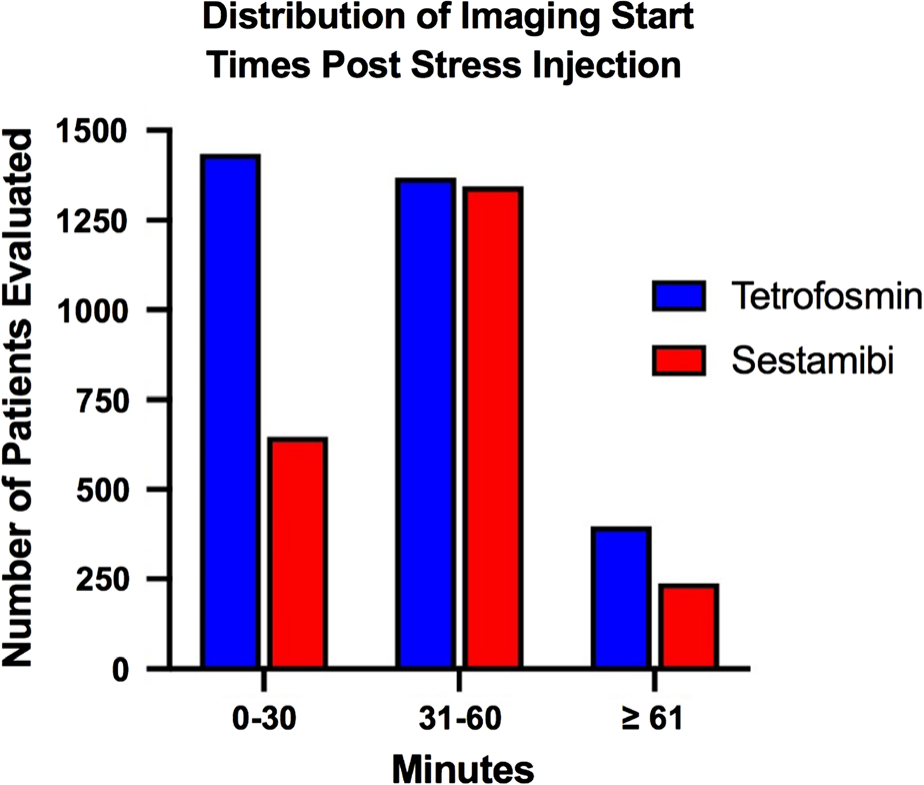
A comparison of the number of patients evaluated based on the time from injection to imaging

Examination of the composite of the studies involving tetrofosmin and sestamibi revealed most studies were designed with an earlier start time for tetrofosmin (Figure 4). In addition, four of the six studies assessing early and late imaging times for tetrofosmin alone demonstrated no difference in the image quality when an earlier start time was used, while two of the studies demonstrated a higher quality with the later start time (Table 3).

**Table 3:** Studies identified with a total of 588 patients assessing image quality comparing early tetrofosmin to late tetrofosmin acquisition

Author	# of patients	Intervention	Early tetrofosmin image quality higher	Equivalent image quality	Delayed tetrofosmin image quality higher
Giorgetti et al.^14^	120	15 vs 45–60 min			
Albutaihi et al.^15^	49	15 vs 45 min			
Philippe et al.^16^	194	Early vs 30 min			
Pirich et al.^17^	97	14 vs 45 min			
Dizdarevic et al.^18^	50	15 vs 45 min			
Katsikis et al.^19^	78	15 vs 45 min			

The size of the diamonds is proportional to the number of patients analyzed in each study

### Comparison of Re-scan Rates Between Tetrofosmin and Sestamibi

Only one study measured real-world endpoints relevant to the efficiency of a nuclear cardiology laboratory such as the re-scan rate and total length of the study. Ravizzini et al imaged 686 patients on two alternating weeks with tetrofosmin or sestamibi over eight months, totaling 1,134 imaging studies.^20^ Tetrofosmin patients were imaged 30 minutes after rest injection, 20 minutes after exercise stress, and 30 minutes after pharmacologic stress while sestamibi patients were imaged at 60, 30, and 45 minutes, respectively. The study found that the total duration of the rest-stress study was 34 minutes shorter (27%) with tetrofosmin compared to sestamibi (90 ± 32.7 vs 124 ± 37, *P* < 0.0001). The re-scan rate due to excessive liver or bowel activity (based on the decision of the interpreting physician who was blinded to the isotope used) was less for tetrofosmin than sestamibi for rest, stress, and combined rest and stress images which reached statistical significance for rest (10.0% vs 21.4%, *P* = 0.001) and combined rest and stress (7.9% vs 19.7%, *P* = 0.01) (Table 4). This decrease in the re-scan rate was in spite of the shorter time from injection to imaging in the tetrofosmin cohort.

**Table 4:** A total of 686 patients imaged with tetrofosmin 30 min after rest injection, 20 min after exercise stress, and 30 min after pharmacologic stress compared to 614 sestamibi patients imaged at 60, 30 and 45 min, respectively assessed for re-scan rates and total study length^20^

	Sestamibi	Tetrofosmin	Difference	*P* value
Re-scan rate				
Rest scan	21.4%	10.0%	11.4%	0.001
Stress scan	9.9%	5.8%	4.1%	0.082
Rest and stress scans	19.7%	7.9%	11.8%	0.01
Time injection to acquisition (min)				
Rest imaging	74.3 ± 25.8	47.7 ± 21.7	26.6	< 0.0001
Stress imaging	48.4 ± 25	42.9 ± 23.3	5.5	< 0.0066
Total imaging	124 ± 37	90 ± 32.7	34	< 0.0001

### Comparison of Heart to Extra-cardiac Uptake

A potential measure of the contrast to noise ratio in myocardial perfusion imaging has been the assessment of myocardial counts compared to counts in adjacent regions such as the lung, liver, or subdiaphragmatic region. A number of studies measure these ratios as a surrogate for imaging quality.

#### Comparison of tetrofosmin and sestamibi start times differences: Heart to extra-cardiac ratios

**Figure 5:**
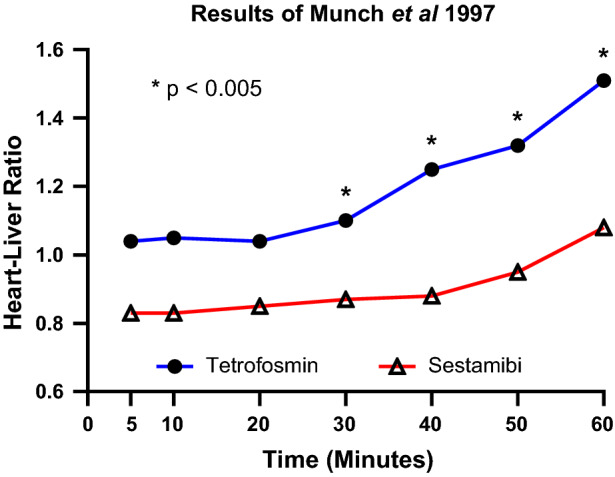
Comparison of heart-to-liver ratios between sestamibi and tetrofosmin imaging at different time points in 24 patients. Adapted from Munch et al.^21^

An early study by Munch et al included 24 patients, 12 imaged with sestamibi and 12 with tetrofosmin in a one-day rest/stress protocol utilizing exercise stress.^21^ Planar images were obtained 5, 10, 20, 30, 40, 50, and 60 minutes following the stress injection and used to calculate heart-liver and heart-lung ratios. They found better myocardial uptake in normal myocardium with tetrofosmin than with sestamibi at stress at all time points (5 minute: 0.37 vs 0.23 [*P* = 0.008]; 60 minute: 0.32 vs 0.22 [*P* = 0.04] counts/pixel). The decay-corrected biological cardiac half-life for tetrofosmin was significantly lower (*P* = 0.008) than sestamibi. Liver uptake of tetrofosmin was initially higher than sestamibi (0.41 vs 0.31) but by 40 minutes was not significantly different than sestamibi. The decay-corrected biological liver half-life for sestamibi was significantly longer than tetrofosmin (136 vs 67 minutes, *P* = 0.02). There was no significant difference at any time point in the heart-lung ratios between the two tracers. Tetrofosmin had a higher heart-liver ratio at all time points (1.04 ± 0.24 at 5 minutes and 1.51 ± 0.44 at 60 minutes vs 0.83 ± 0.16 at 5 minute and 1.08 ± 0.27 at 60 minutes) which met statistical significance (*P* < 0.05) at 30 minutes and later (Figure 5).

In a study of 32 patients who underwent an exercise-rest one-day SPECT MPI study with both tetrofosmin and sestamibi within a week, imaging with sestamibi was performed 60 minutes after tracer injection while there was only a 30 minute delay with tetrofosmin.^10^ The heart/lung and heart/liver ratios were derived from the anterior image of the tomographic acquisition at this time and compared. The heart-liver ratios were similar between tetrofosmin and sestamibi at both rest (0.93 vs 0.86) and stress (1.20 vs 1.16) as were the heart-lung ratios. Similar ratios with tetrofosmin imaging performed 30 minutes earlier support similar delineation of the heart much earlier.

Hurwitz et al investigated early (15 to 30 minutes) and late (30 to 60 minutes) image acquisition with tetrofosmin and sestamibi in 107 patients.^11^ Sestamibi had 20-25% higher normalized myocardial counts than tetrofosmin after stress and rest imaging regardless of the timing. Sestamibi had non-statistically significant lower ratios of heart-adjacent background activity to heart activity compared to tetrofosmin which reached statistical significance when the greatest ratios of background activity in the field of view (usually GI activity) to heart activity were compared. In general, the background-heart ratios were worse with early compared to late imaging and rest compared to stress.

In the large, multi-center study of 2,523 patients imaged with tetrofosmin, sestamibi, or thallium by Kapur et al, tetrofosmin imaging was performed on average 10 minutes earlier than sestamibi (40 minutes vs 49 to 50 minutes).^12^ The anterior planar image of the tomographic acquisition was used to measure counts in the myocardium, lung, liver, and subdiaphragmatic region. There were significantly greater counts in the heart, lung, liver, and subdiaphragmatic region with sestamibi compared to tetrofosmin for stress images, but only for the lung and subdiaphragmatic region for rest images. However, despite the 10-minute earlier imaging with tetrofosmin, there was no significant difference between the two tracers, at stress and rest, in heart-to-subdiaphragmatic, -lung, or -liver ratios.

A multi-center study which enrolled 260 patient acquisitions (dipyridamole stress and rest images) compared sestamibi to tetrofosmin at different imaging delays (0.5, 1, and 2 hours).^22^ At the same time it also assessed the effect of ingesting milk, water, or nothing after the isotope injection. Myocardial counts and an extra-cardiac region of interest immediately below the myocardium were calculated using the raw anterior projection. While this myocardial-to-extra-cardiac ratio improved with delayed imaging, the ratios were not statistically different between the sestamibi and tetrofosmin (*P* = 0.42).

A small study by Turgut et al in 2005 with 19 patients employed a one-day rest-dobutamine stress performed with both sestamibi and tetrofosmin one week apart.^23^ This was one of the few studies to image with tetrofosmin and sestamibi in the same patient. The injection to image acquisition time for sestamibi was 60 minutes for rest and stress and 30 minutes for tetrofosmin. A dedicated 5 minute anterior planar acquisition was used to acquire heart, lung, and liver counts. The authors reported that the heart-to-liver or lung ratios were not statistically different between tetrofosmin and sestamibi in all patients nor in those patients with and without coronary artery disease.

**Figure 6:**
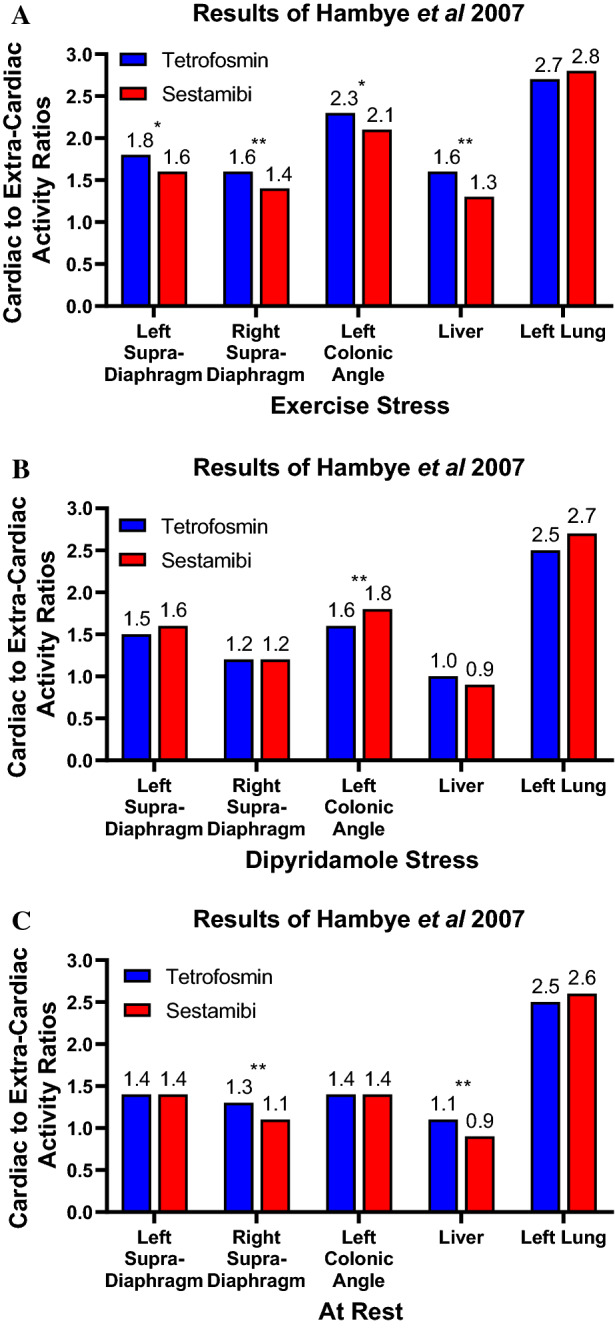
Comparison of sestamibi and tetrofosmin ratios in Hambye et al evaluating 425 patients.^13^ Statistically significant *P* values are noted (*0.01 < *P* < 0.05; **P < 0.01)

In the study by Hambye et al with 425 consecutive patients alternating imaging with either tetrofosmin or sestamibi there were no significant differences in the time from injection to imaging for rest and exercise stress; however, with pharmacologic stress sestamibi imaging occurred 27 minutes later (*P* = 0.02).^13^ The anterior planar view was used to create a myocardial region of interest as well as five extra-cardiac regions. There was no statistically significant difference in myocardial activity with exercise stress (0.21 vs 0.20, *P* = 0.35) between sestamibi and tetrofosmin or for dipyridamole stress (0.21 vs 0.19, *P* = 0.32), but a difference was seen with rest images (0.21 vs 0.016, *P* = 0.0003). Most of the cardiac/extra-cardiac ratios were higher with tetrofosmin, both after rest and exercise stress, as compared to sestamibi. However, variable results were observed after dipyridamole induced stress in which sestamibi was imaged 27 minutes later than tetrofosmin (Figure 6).

Of the seven studies included in this analysis, five demonstrated no difference between heart and extra-cardiac ratios between sestamibi and tetrofosmin despite using significantly earlier starting times with tetrofosmin, and in two studies, the heart-to-liver ratios were higher with tetrofosmin (Table 5).

**Table 5:** Studies identified with a total of 3,390 patients assessing heart to extra-cardiac ratios comparing sestamibi to tetrofosmin image acquisition, often with the tetrofosmin acquisition occurring early. The only significant difference in a larger study (Hambye et al)^13^ indicated an improved heart-to-liver ratio when sestamibi and tetrofosmin at rest using a similar start time

Author	# of patients	Intervention (min)		Sestamibi higher heart to extra-cardiac ratios	Equivalent heart to extra-cardiac ratios	Tetrofosmin higher heart to extra-cardiac ratios
		Sestamibi	Tetrofosmin			
Munch et al.^21^	24	5, 10, 20, 30, 40, 50, 60				
Acampa et al.^10^	32	60	30			
Hurwitz et al.^11^	107	30-60	15-30			
Kapur et al.^12^	2,523	50	40			
Peace et al.^22^	260	30, 60, 120				
Turgut et al.^23^	19	60	30			
Hambye et al.^13^	425	30	Early			

The size of the diamonds is proportional to the number of patients analyzed in each study

#### Comparison of tetrofosmin start times differences: Heart to extra-cardiac ratios

In 1998, Mann et al studied 106 patients injected at rest with tetrofosmin during an acute chest pain protocol with imaging occurring 15 to 270 minutes after injection.^24^ Patients were placed into 5 groups based on acquisition time after injection (15 to 30 minutes, 31 to 45 minutes, 46 to 60 minutes, 60 to 90 minutes, and > 90 minutes) and the heart-liver ratios were measured. The 15 to 45-minute groups had a mean ratio ≤ 1.0 and the ratios of the groups over 46 minutes were significantly higher than those of shorter duration. Unfortunately, there was no sestamibi comparison group.

Giorgetti et al evaluated 120 patients comparing tetrofosmin at two imaging delay times (15 minutes vs 45 minutes).^14^ Anterior raw images were used to calculate counts in the myocardium, lungs, liver and subdiaphragmatic area including the liver. The authors reported no significant differences in heart, lung, liver, and subdiaphragmatic counts between the two times after tetrofosmin injection (76, 43, 115, 76 vs. 77; 42; 104, 81 counts/pixel for 15 and 45 minutes, respectively). While there was negligible washout from the heart, lung, and subdiaphragmatic area, there was a 5% washout in the liver between the early and late imaging times.

In the study by Philippe et al 194 patients were imaged with tetrofosmin early post-stress, and 30 minutes post-stress.^16^ The exact time of the early post-stress imaging was not defined. Single-frame anterior projections were used to acquire the counts in the myocardium, liver, and lung. The authors reported significant differences in the cardiac to lung-liver ratios between tetrofosmin early and 30 minutes post-stress with ratios increasing with increased imaging time.

**Figure 7:**
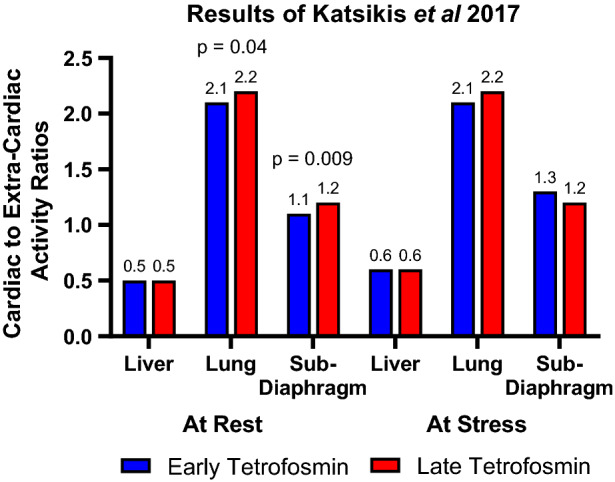
Comparison of heart-to-liver,-lung, and -subdiaphragmatic area activity ratios between early and late tetrofosmin imaging in 78 patients.^19^ Statistically significant *P* values are noted

In another study of 78 patients that were imaged with tetrofosmin early and late (15 and 45 minutes) after stress and rest injection, the anterior raw images were used to create regions of interest over the heart, lungs, liver, and subdiaphragmatic area.^19^ The stress and rest counts in the heart, liver, lungs, and subdiaphragmatic areas were all greater at 15 minutes. However, there was no significant difference in any of the stress heart-to-anything ratios. For rest images, there was a statistically significant increase in the heart-lung (2.1 vs 2.2, *P* = 0.04) and heart-subdiaphragmatic area (1.1 vs 1.2, *P* = 0.009) ratios comparing early to late imaging delay times (Figure 7).

Of the 4 studies examined, two studies demonstrated improvement in the heart to extra-cardiac ratios with increasing time to acquisition, while two reported no change (Table 6).

**Table 6:** Studies identified with a total of 498 patients assessing heart to extra-cardiac ratios comparing early tetrofosmin to late tetrofosmin acquisition

Author	# of patients	Intervention	Early tetrofosmin higher heart to extra-cardiac ratios	Equivalent heart to extra-cardiac ratios	Delayed tetrofosmin higher heart to extra-cardiac ratios
Mann et al.^24^	106	15 to >90 min			
Giorgetti et al.^14^	120	15 vs 45 min			
Philippe et al.^16^	194	15 vs 45 min			
Katsikis et al.^19^	78	15 vs 45 min			

The size of the diamonds is proportional to the number of patients analyzed in each study

## Discussion

The efficient performance of diagnostic testing has always been a significant consideration for the patient experience and overall functioning of the imaging laboratory, but it has grown in importance in the current cost-conscious world of comparative effectiveness of testing modalities. A rest-stress SPECT MPI study is one of the longest studies for the diagnosis or assessment of coronary artery disease when compared to competitive modalities such as cardiac PET MPI, stress echocardiography, CT coronary angiography, and even invasive coronary angiography.^25^ The efficient throughput of patients in the nuclear cardiology laboratory has a direct effect on quality metrics such as hospital length of stay, patient satisfaction and the efficient use of equipment and staff time. The imaging delay time between injection of radioisotope and the start of rest or stress imaging represents a significant inefficiency in SPECT MPI testing, and the two or three-fold increase in this duration has a negative impact on the patient experience and overall time of testing. In addition, sub-optimal or non-diagnostic images obtained after too short of an imaging delay can substantially increase testing time by having to wait additional time and repeat imaging. The need for repeat imaging creates havoc with patient throughput, introduces unnecessary delays to other patients and decreases the overall efficient use of SPECT systems. In the future, with the development of SPECT quantification of absolute coronary flow and coronary flow reserve, images will need to be acquired during vasodilator stress hyperemia.^26^ The imaging characteristics seen with early tetrofosmin imaging at 15 minutes demonstrate a flexibility in imaging that can have a direct impact on laboratory efficiency, patient satisfaction and potentially could enable SPECT flow quantification.^27^

Overall, the data reviewed show that earlier imaging with tetrofosmin is equivalent to later imaging with sestamibi or tetrofosmin when assessing subjective image quality or when quantifying heart-to-extra-cardiac ratios. The equivalency of the imaging quality occurs with 15 minutes (on average) earlier imaging compared to sestamibi and 30 minutes compared to standard time tetrofosmin. The basis for improved image quality and less subdiaphragmatic uptake with tetrofosmin permitting earlier imaging is based on a small amount of renal clearance and faster washout from the liver.^28^ Of the four studies which examined using image quality as the endpoint with head-to-head comparisons of sestamibi and tetrofosmin with tetrofosmin being imaged earlier than sestamibi, two of the studies were unable to detect a difference in image quality between sestamibi and tetrofosmin. Additionally, in one study tetrofosmin was rated as superior, while in the other, sestamibi was rated superior. Only one of these studies including 32 patients, Acampa et al.,^10^ studied both tracers in the same patient and found no difference in image quality despite a 30 minute longer delay for sestamibi. In four of the six studies using image quality as the endpoint assessing early and late imaging times for tetrofosmin alone, no difference in the image quality was seen when an earlier start time was used, while in the two other studies a higher quality was seen with the later start time. The subjective endpoint of equivalent image quality was also assessed with objective measurements of the surrogate marker of heart-to-extra-cardiac ratios in early versus late imaging. The majority of studies reviewed found no significant difference between heart and extra-cardiac ratios between sestamibi and tetrofosmin despite using significantly earlier starting times with tetrofosmin. In the only study assessing the two tracers in the same patient, Turgut et al.,^23^ an evaluation of 19 patients found no difference in heart to extra-cardiac ratios despite a 30 minute shorter imaging delay for tetrofosmin. Meta-analysis could not be accurately performed on these groups of articles due to the heterogenous nature of the studies, variability in methodology, and non-uniform endpoints.

Perhaps the one study to look at a practical real-world endpoint assessed the re-scan rate and total length of the MPI study.^20^ Tetrofosmin patients were imaged 10-30 minutes earlier than sestamibi patients depending on the stressor or phase of imaging, and study length was found to be 27% shorter with tetrofosmin, which was a statistically and clinically significant 34 minutes shorter. The re-scan rate due to excessive liver or bowel activity was less for tetrofosmin than sestamibi, most notably for rest images, which translated into a reduction in the re-scan rate for the entire rest-stress study duration. These real-world endpoints are the practical consequence of the heart-to-extra-cardiac ratios and image quality differences seen in the other studies.

When selecting search criteria for this review, diagnostic accuracy between sestamibi and tetrofosmin was not investigated due to small sample size, incomplete data and referral bias in patients with coronary angiography thus preventing a valid comparison. Still a number of papers identified by the methodology of this systematic review contained endpoints attempting to assess the diagnostic accuracy of the perfusion results. Due to the biases implicit in these analyses, the accuracy of the perfusion results at different imaging delay times could not be reviewed. Out of necessity, non-English language articles were excluded from the analysis, although no significant contributions to the literature were felt to have been missed. In regards to only 47% of the studies having a low risk of bias and low concern regarding applicability in the reference standard domain portion of the QUADAS-2 tool applied to the articles in the review, this has few implications for the results comparing the two tracers (tetrofosmin versus sestamibi) or the different image to acquisition times (early versus late) as the results will be relative to each other not compared to a reference standard. This lower rating would be of concern if diagnostic accuracy in terms of sensitivity and specificity compared to a reference standard such as coronary angiography was being assessed for a single group and was another rational as to why it was not performed.

## Conclusion

The composite of this data shows that earlier imaging with tetrofosmin is equivalent to later imaging with sestamibi when assessing subjective image quality or when quantifying heart-to-extra-cardiac ratios. Image quality and heart-to-extra-cardiac ratios comparing early versus later imaging with tetrofosmin were comparable if not equivalent to each other. The equivalency of the imaging quality occurs with 15 minutes (on average) earlier imaging compared to sestamibi and 30 minutes compared to standard time tetrofosmin. The subjective findings of equivalent image quality are also shown with objective measurements of heart-to-extra-cardiac ratios. Clinically these imaging findings translated into improved clinical laboratory efficiency with tetrofosmin with decreased need for re-scans and shorter test completion time in the one study examining laboratory performance outcomes.^20^ In this study, the significantly shorter injection-to-acquisition times with tetrofosmin compared to sestamibi resulted in better efficiency and less waiting times for patients; in addition, significantly higher re-scan rates with sestamibi compared to tetrofosmin due to hepatic activity contributed to better throughput with tetrofosmin.

## Electronic supplementary material

Below is the link to the electronic supplementary material.

Supplementary material 1 (PPTX 190 kb)

## Abbreviations

SPECT: Single photon emission computed tomography
PET: Positron emission tomography
MPI: Myocardial perfusion imaging
ASNC: American Society of Nuclear Cardiology
QUADAS: Quality Assessment Tool for Diagnostic Accuracy guidelines
