# Supplementary material for: Efficiency of tetrofosmin versus sestamibi achieved through shorter injection-to-imaging times: A systematic review of the literature
Source: J Nucl Cardiol. 2020 Mar 31;28(4):1381–94. doi: 10.1007/s12350-020-02093-5 (PMC8421307; doi:10.1007/s12350-020-02093-5)
Supplement: Supplementary file 1 — Supplementary material 1 (PPTX 190 kb) [file 12350_2020_2093_MOESM1_ESM.pptx]

## Slide 1
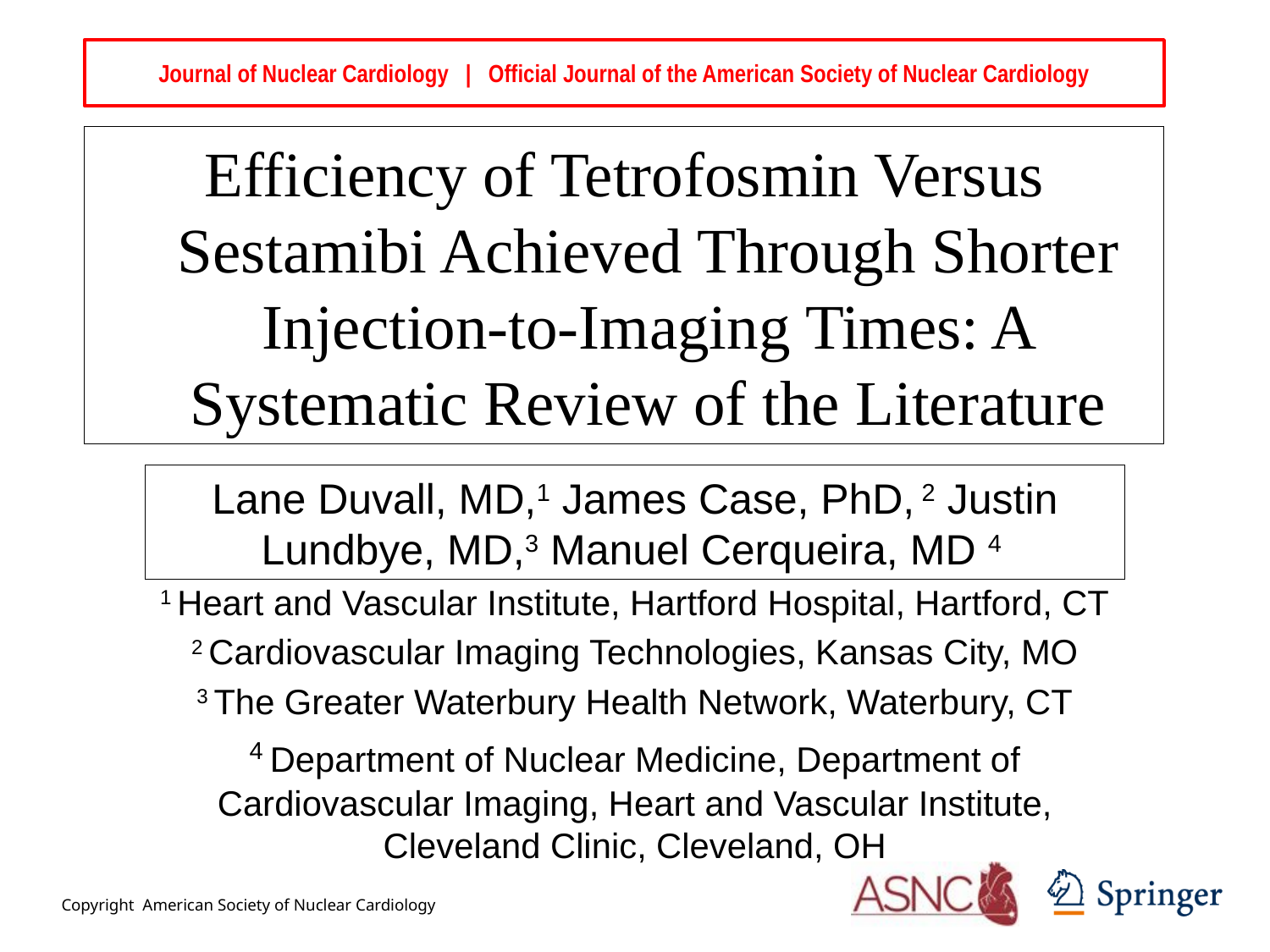

Journal of Nuclear Cardiology | Official Journal of the American Society of Nuclear Cardiology
# Efficiency of Tetrofosmin Versus Sestamibi Achieved Through Shorter Injection-to-Imaging Times: A Systematic Review of the Literature
Lane Duvall, MD,1 James Case, PhD, 2 Justin Lundbye, MD,3 Manuel Cerqueira, MD 4
1 Heart and Vascular Institute, Hartford Hospital, Hartford, CT
2 Cardiovascular Imaging Technologies, Kansas City, MO
3 The Greater Waterbury Health Network, Waterbury, CT
4 Department of Nuclear Medicine, Department of Cardiovascular Imaging, Heart and Vascular Institute, Cleveland Clinic, Cleveland, OH
Copyright American Society of Nuclear Cardiology

## Slide 2
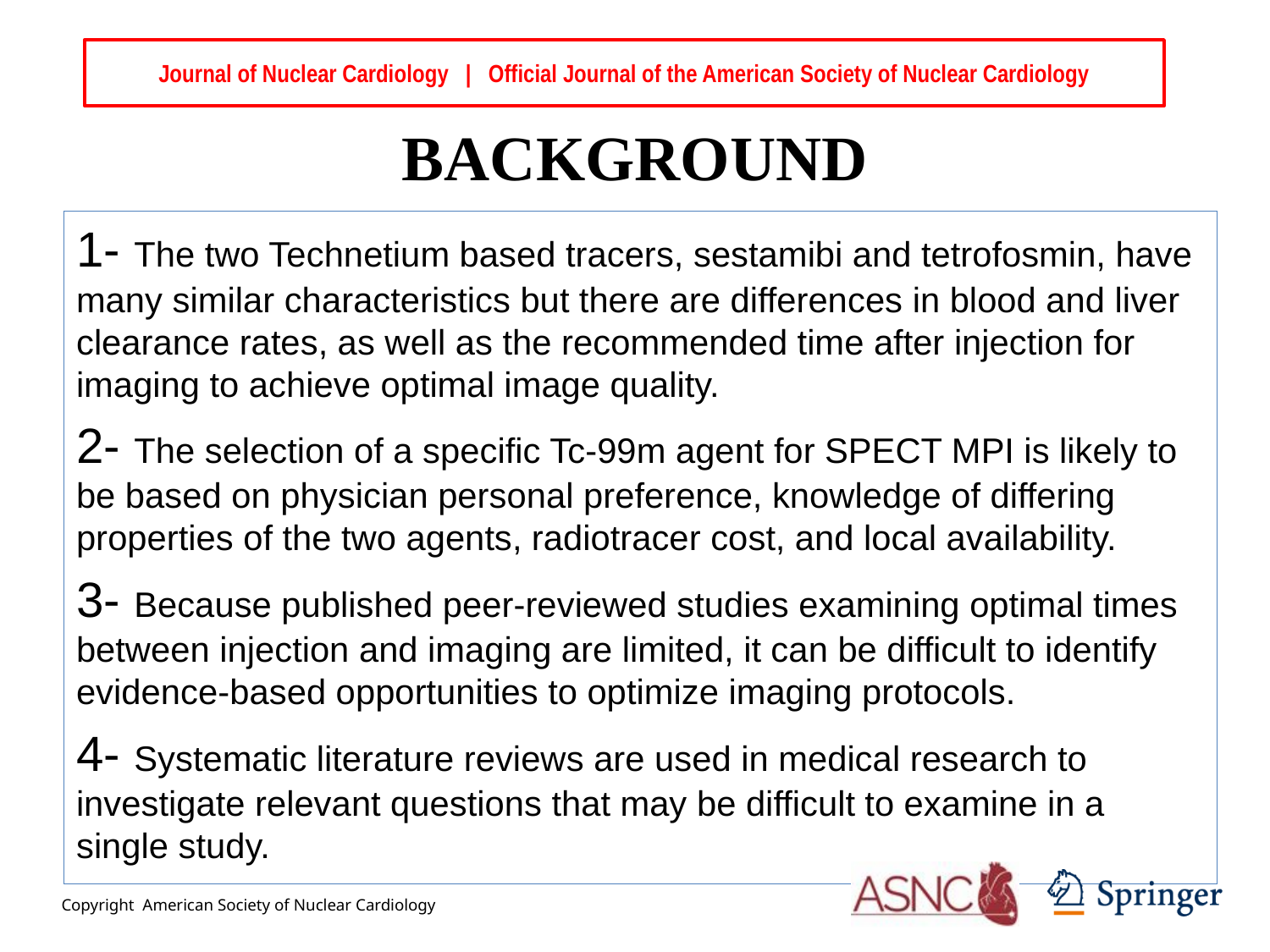

Journal of Nuclear Cardiology | Official Journal of the American Society of Nuclear Cardiology
# BACKGROUND
1- The two Technetium based tracers, sestamibi and tetrofosmin, have many similar characteristics but there are differences in blood and liver clearance rates, as well as the recommended time after injection for imaging to achieve optimal image quality.
2- The selection of a specific Tc-99m agent for SPECT MPI is likely to be based on physician personal preference, knowledge of differing properties of the two agents, radiotracer cost, and local availability.
3- Because published peer-reviewed studies examining optimal times between injection and imaging are limited, it can be difficult to identify evidence-based opportunities to optimize imaging protocols.
4- Systematic literature reviews are used in medical research to investigate relevant questions that may be difficult to examine in a single study.
Copyright American Society of Nuclear Cardiology

## Slide 3
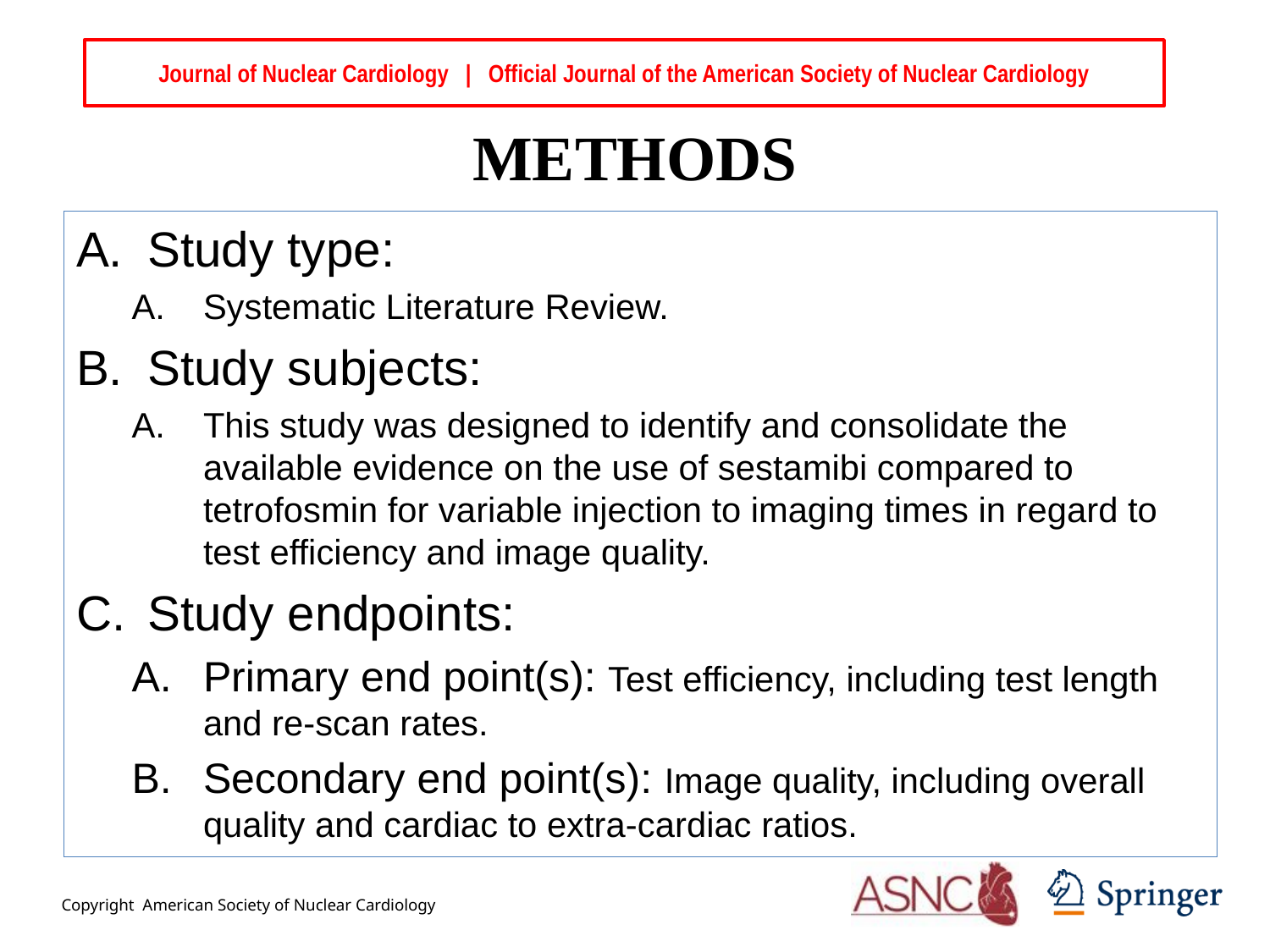

Journal of Nuclear Cardiology | Official Journal of the American Society of Nuclear Cardiology
# METHODS
Study type:
Systematic Literature Review.
Study subjects:
This study was designed to identify and consolidate the available evidence on the use of sestamibi compared to tetrofosmin for variable injection to imaging times in regard to test efficiency and image quality.
Study endpoints:
Primary end point(s): Test efficiency, including test length and re-scan rates.
Secondary end point(s): Image quality, including overall quality and cardiac to extra-cardiac ratios.
Copyright American Society of Nuclear Cardiology

## Slide 4
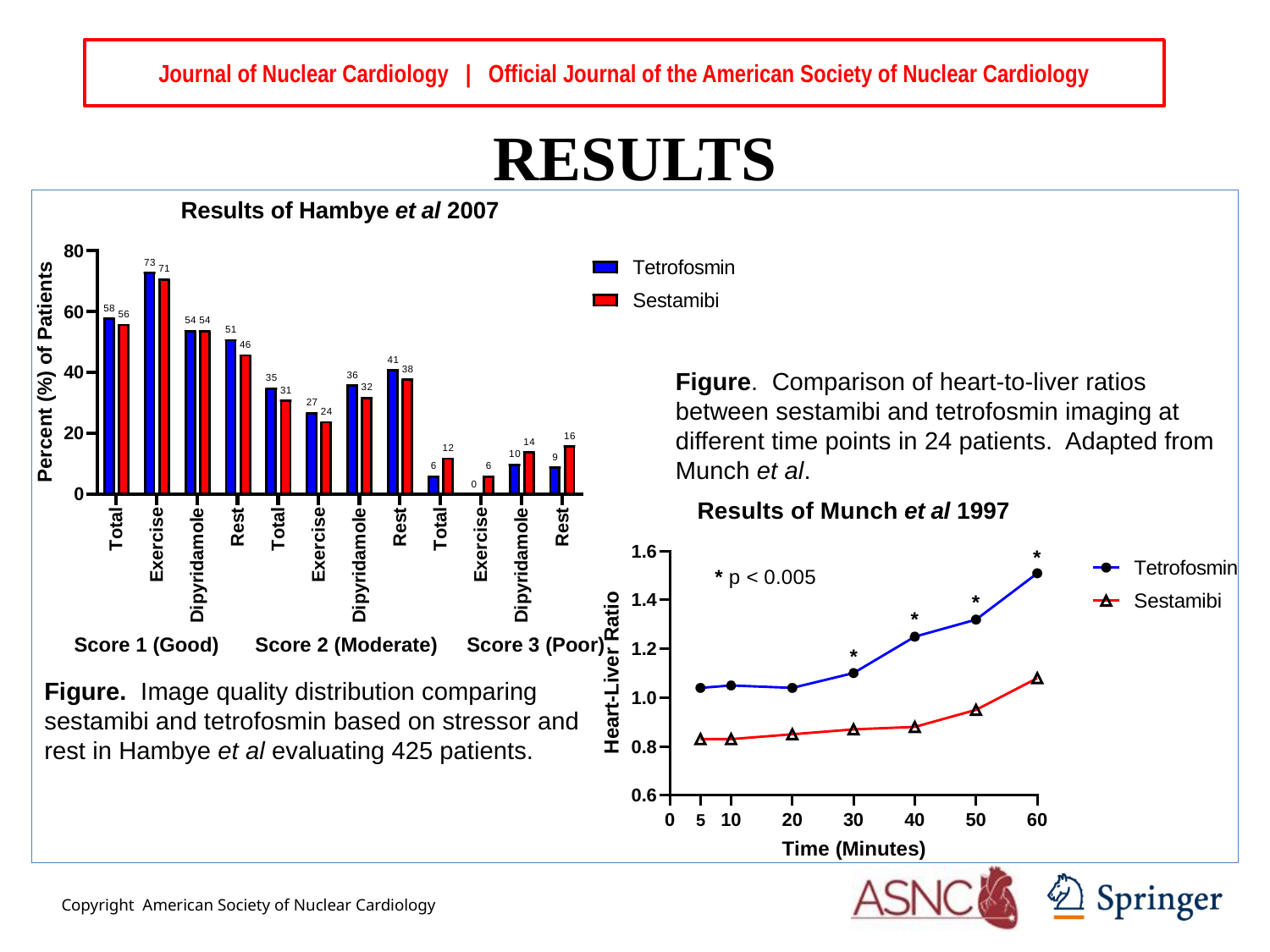

Journal of Nuclear Cardiology | Official Journal of the American Society of Nuclear Cardiology
# RESULTS
Figure. Comparison of heart-to-liver ratios between sestamibi and tetrofosmin imaging at different time points in 24 patients. Adapted from Munch et al.
Figure. Image quality distribution comparing sestamibi and tetrofosmin based on stressor and rest in Hambye et al evaluating 425 patients.
Copyright American Society of Nuclear Cardiology

## Slide 5
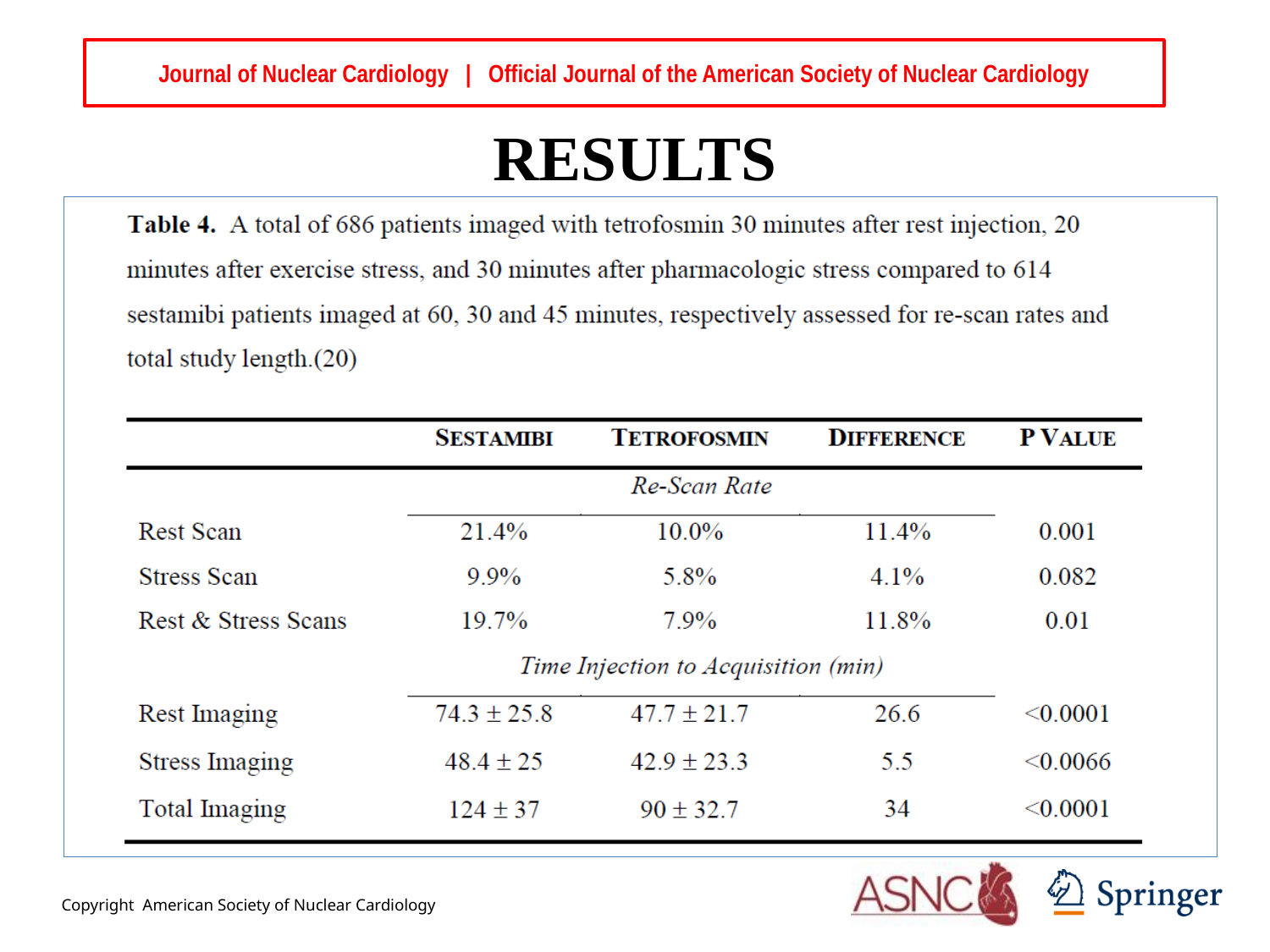

Journal of Nuclear Cardiology | Official Journal of the American Society of Nuclear Cardiology
# RESULTS
Copyright American Society of Nuclear Cardiology

## Slide 6
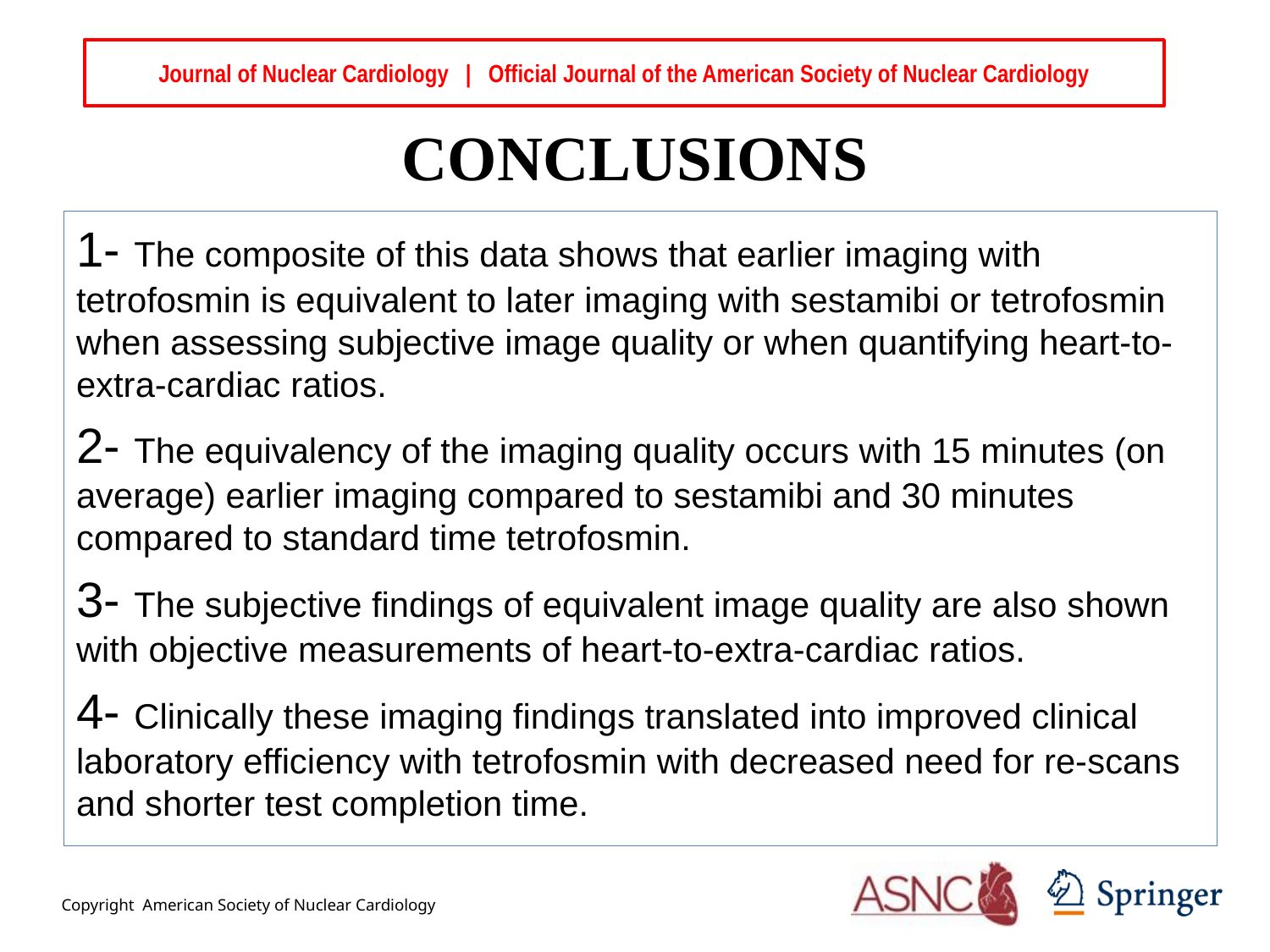

Journal of Nuclear Cardiology | Official Journal of the American Society of Nuclear Cardiology
# CONCLUSIONS
1- The composite of this data shows that earlier imaging with tetrofosmin is equivalent to later imaging with sestamibi or tetrofosmin when assessing subjective image quality or when quantifying heart-to-extra-cardiac ratios.
2- The equivalency of the imaging quality occurs with 15 minutes (on average) earlier imaging compared to sestamibi and 30 minutes compared to standard time tetrofosmin.
3- The subjective findings of equivalent image quality are also shown with objective measurements of heart-to-extra-cardiac ratios.
4- Clinically these imaging findings translated into improved clinical laboratory efficiency with tetrofosmin with decreased need for re-scans and shorter test completion time.
Copyright American Society of Nuclear Cardiology
